# Biomarkers and Stimulation Algorithms for Adaptive Brain Stimulation

**DOI:** 10.3389/fnins.2017.00564

**Published:** 2017-10-10

**Authors:** Kimberly B. Hoang, Isaac R. Cassar, Warren M. Grill, Dennis A. Turner

**Affiliations:** ^1^Department of Neurosurgery, Duke University, Durham, NC, United States; ^2^Department of Biomedical Engineering, Duke University, Durham, NC, United States; ^3^Department of Neurobiology, Duke University Medical Center, Duke University, Durham, NC, United States

**Keywords:** deep brain stimulation, epilepsy, Parkinson's disease, responsive brain stimulation, beta hypersynchrony, phase amplitude coupling, evoked field potentials, closed loop

## Abstract

The goal of this review is to describe in what ways feedback or adaptive stimulation may be delivered and adjusted based on relevant biomarkers. Specific treatment mechanisms underlying therapeutic brain stimulation remain unclear, in spite of the demonstrated efficacy in a number of nervous system diseases. Brain stimulation appears to exert widespread influence over specific neural networks that are relevant to specific disease entities. In awake patients, activation or suppression of these neural networks can be assessed by either symptom alleviation (i.e., tremor, rigidity, seizures) or physiological criteria, which may be predictive of expected symptomatic treatment. Secondary verification of network activation through specific biomarkers that are linked to symptomatic disease improvement may be useful for several reasons. For example, these biomarkers could aid optimal intraoperative localization, possibly improve efficacy or efficiency (i.e., reduced power needs), and provide long-term adaptive automatic adjustment of stimulation parameters. Possible biomarkers for use in portable or implanted devices span from ongoing physiological brain activity, evoked local field potentials (LFPs), and intermittent pathological activity, to wearable devices, biochemical, blood flow, optical, or magnetic resonance imaging (MRI) changes, temperature changes, or optogenetic signals. First, however, potential biomarkers must be correlated directly with symptom or disease treatment and network activation. Although numerous biomarkers are under consideration for a variety of stimulation indications the feasibility of these approaches has yet to be fully determined. Particularly, there are critical questions whether the use of adaptive systems can improve efficacy over continuous stimulation, facilitate adjustment of stimulation interventions and improve our understanding of the role of abnormal network function in disease mechanisms.

## Introduction

Current evidence points to various forms of invasive brain stimulation, including stimulation for epilepsy and deep brain stimulation (DBS) for movement disorders, as exerting widespread influence over multiple brain areas through modulation of disease- and patient-specific neural networks (Zamora-Lopez et al., 2011; Henderson, 2012; Lozano and Lipsman, 2013; Fox et al., 2014; Horn et al., 2017). These dynamic networks may be partially identified by structural or axonal connectivity (i.e., as demonstrated by diffusion tensor imaging tractography) or functional and physiological connectivity [i.e., as demonstrated by positron emission tomography, functional magnetic resonance imaging (MRI)] (Oswal et al., 2016). However, clinical targeting is typically confirmed by direct electrophysiological recordings and or macrostimulation to evoke the desired symptom response. Precise placement of electrodes to interact with specific brain networks is currently initially guided by pre-operative recordings (particularly in epilepsy) and imaging, with detailed MRI to define approximate anatomical localization due to high patient to patient variability. Further, in many indications microelectrode recording to refine the optimal physiological subregion is critical, since this region may be distinct from the initial anatomical target. Finally, engagement of appropriate circuitry within the desired network can be verified by clinical assessment. During awake DBS procedures, for example, appropriate neural network engagement can be directly confirmed by symptom suppression (relief of tremor, for example). The predicted sphere of activation or influence of stimulation (Butson et al., 2011) may assist with targeting. However, clinical assessment remains the primary verification of network activation in use at this time.

Although continuous DBS has significantly improved the treatment of Parkinson's disease (Odekerken et al., 2016) and tremor, this mode of stimulation has many limitations and has had limited success in other diseases, such as depression or Alzheimer's disease. The current clinical approach to programming and adjusting stimulation parameters is time consuming and a more automated approach is desirable. Likewise, improved power efficiency and fewer side effects would improve clinical treatment. Many of these improvements will be facilitated by identification and development of biomarkers linked to both network activity and symptom relief, if these relevant biomarkers can be successfully integrated into the treatment scheme. Thus, possible improvements in care may be achieved, including definitively mapping electrode location during surgical procedures (in addition to symptom suppression or as a separate, objective marker), continuous, dynamic adjustment of stimulation in either an amplitude- or time-dependent manner (Rosa et al., 2015), improved control of disease symptoms (Tinkhauser et al., 2017), and also enhancement of device lifespan, through intermittent or reduced stimulation. Beyond the operative environment, biomarkers may also facilitate initial clinical programming to provide objective endpoints as well as facilitate long-term automatic adjustment (Heldman et al., 2016). Additionally, biomarkers may help provide insight into the treatment efficacy variability between patients with optimal electrode position and long-term disease management (Trager et al., 2016). However, such insight will require confirming a direct correlation between the biomarkers reflecting underlying neural network activity and associated clinical symptoms (Kuhn et al., 2006, 2009). Confirmation of engagement of relevant neural circuitry may also help to understand possible divergence between patient-specific anatomical targeting and physiological activation of circuits.

Additional techniques under development include novel stimulation paradigms, which may be more clinically efficacious. These include structured stimulation patterns developed from extensive modeling of Parkinson's circuitry, tested intraoperatively, now being assessed in clinical trials, which include variable stimulation pulse timing (rather than regular pulse generation) and can potentially reduce the number of pulses per second needed for effective stimulation (Brocker et al., 2017), while also potentially improving the efficiency of stimulation (Brocker et al., 2013). Further, novel electrode designs are now just coming into clinical practice, including 8 contact directional electrodes (Steigerwald et al., 2016; Volkmann et al., 2016) and a research design with additional contacts spaced around the electrode (Contarino et al., 2014), both allowing for current to be steered toward treatment areas or away from critical regions. Both of these advances could also be used in combination with various types of responsive or adaptive control systems, to implement a hybrid system with multiple improved techniques.

Possible biomarkers span multiple modalities, including external wearable devices (such as an accelerometer or step counter) that analyze a symptom and may communicate with a common controller (Graupe et al., 2010; Shull et al., 2014; Ekker et al., 2016). Further, internal markers of circuitry function have been suggested, such as beta frequency oscillatory activity (Silberstein et al., 2003) and associated phase amplitude coupling (PAC) (De Hemptinne et al., 2015). Evoked field potentials arising from the DBS stimulation pulses (Kent et al., 2015) and triggering on intermittent events could be used for initiating or adjusting stimulation (Fisher and Velasco, 2014) or Tourette's (Almeida et al., 2015). Biomarkers could also be biochemical changes detected by a sensor (i.e., dopamine) (Grahn et al., 2014), blood flow (Hill et al., 2013; Haense et al., 2016; Noor et al., 2016), temperature changes, or optogenetic signals (Bernstein et al., 2012). Identification of appropriate biomarkers will be critical in the execution of responsive on/off triggerable systems (with a fixed, preset response to a threshold input), adaptive (with adjustable duration or scalar, graded adjustments in amplitude to reach a pre-defined setpoint) or dynamic, closed-loop feedback systems with multiple inputs. All of these formats require some form of circuitry-dependent signal relevant to the disease or condition for automatic adjustment of DBS settings (Carron et al., 2013; Little et al., 2013; Rehan and Hong, 2013; Meidahl et al., 2017).

Here we review the design of responsive, adaptive and closed-loop stimulation. We then discuss how various biomarkers and devices may be useful to improve stimulation to treat selected example disease conditions, some of which have already reached the market (i.e., responsive Neuropace device for epilepsy treatment), but mostly which remain experimental or conceptual. As with any new field, the translation of biomarkers into portable or implanted devices will require more critical analysis of the clinical utility in a particular condition, stability over time, the ability to record the signal, and the correlation of the proposed biomarker with clinical symptoms relevant to the disease (Kuhn et al., 2006, 2009; Meidahl et al., 2017). However, few of the biomarkers to be discussed are at this stage of translation yet into humans or clinical trials, hence considerable skepticism remains about introducing more complexity into the already complicated field of brain stimulation (Arlotti et al., 2016). Thus, we mention a number of possible biomarker signals, but as further analysis proceeds and feasibility data are obtained, many of these may or may not prove durable for clinical translation.

## Modes, devices, and control approaches for adaptive stimulation

Stimulation for treatment of epilepsy (using the Neuropace device or anterior thalamic DBS) has just reached clinical usefulness (Fisher and Velasco, 2014), whereas DBS stimulation for movement disorders provides efficacy of ~75% in patients when applied in a continuous, open-loop fashion (for example, Activa SC/PC technology, Medtronic) (Almeida et al., 2015; Odekerken et al., 2016). However, as indications for brain stimulation in other disease processes expand with further knowledge of networks that may benefit from stimulation, additional types of stimulation will likely be needed. In contrast, several clinical trials using DBS for alternative indications (i.e., depression, Alzheimer's disease) have demonstrated less efficacy and remain experimental (Hamani et al., 2009; Dougherty et al., 2015; Lozano et al., 2016). We initially review the various modes of stimulation that have been developed and associated devices, which may be able to sense and deliver adaptive stimulation.

**Figure 1:**
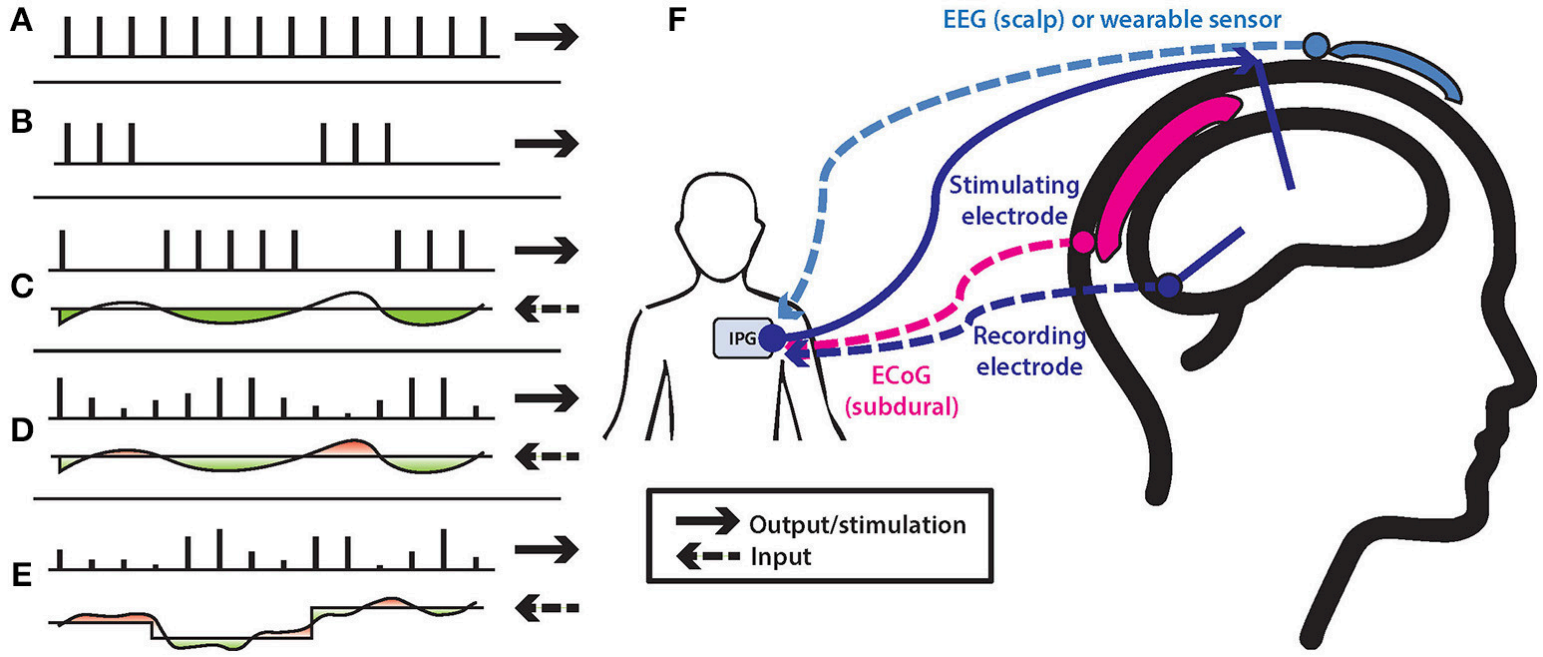
DBS stimulation control models: **(A)** continuous stimulation with evenly spaced stimulation pulses; **(B)** scheduled intermittent stimulation with a burst of pulses at fixed intervals; **(C)** responsive stimulation, with triggered output based on a threshold, but each pulse is the same, preset amplitude; **(D)** adaptive stimulation, with threshold-based adjustments in number of pulses or in amplitude of pulses, settling in around a fixed control point; **(E)** closed-loop stimulation, where the constantly changing, complex input determines an equally complex, dynamic output. For each the solid arrow depicts the output stimulation and the dashed arrow depicts an input signal that is compared to some threshold. **(F)** Potential stimulation and recording arrays. Depending on disease pathology, inputs may be from the DBS lead itself as well as secondary DBS, EEG, or ECoG electrodes. Output stimulation from the IPG is primarily though the DBS/parenchymal lead at this time, although potential exists for subdural and scalp intervention as well.

The idea of a time constant, well-known in control systems literature, is a measure of the time required for the system to respond after a step input, assuming the device can respond faster than the system under observation (Carron et al., 2013). For example, tremor responses to thalamic DBS may take at least 10–20 s to stabilize (Rehan and Hong, 2013; Yamamoto et al., 2013) whereas steady state responses of subthalamic DBS for bradykinesia may occur within a few seconds but require >30 min for stabilization (Waldau et al., 2011). Briefly, control models of stimulation may be broken down into five major subtypes (Figure 1, Table 1), depending on the frequency of the updating needed, the flexibility of stimulation response, and rate of responsiveness. In order of increasing control systems complexity, these include simple continuous on (Figure 1A), intermittent or scheduled with a predetermined schedule and fixed amplitude (Figure 1B), responsive with a triggered onset of a preset amplitude and width (Figure 1C), and adaptive with flexibility during either on/off of a preset amplitude (i.e., variable durations of stimulation) in response to a threshold for a single biomarker input or a variable amplitude (Figure 1D). Finally, in the brain-machine interface context, closed-loop implies a multidimensional input (i.e., multiple spike trains or other input signal denoting the brain's intent), a continuous processing of this input for output predictions, and some form of feedback signal to refine the nearly constant output (Figure 1E). However, any signal where some feedback exists in a control sense could be termed closed loop in a generic sense.

**Table 1:** Control systems description.

**Control type**	**Feedback type**	**Nature of feedback**	**Time constant of activation**
Continuous	Clinician observation	Clinician adjustment	monthly
Scheduled Intermittent	None	Preset stimulation amplitude turned on or off at preset timing	Preset timing determined by system physiology or empirically
Responsive	Triggered by threshold event	Preset stimulation amplitude turned on or off by trigger, with defined lockouts	0.5–5 s, can be repeated
Adaptive	Single biomarker input, continuous monitoring	Stimulation output can be turned on or off, or scaled, based on biomarker input for continuous adjustment	Tremor ~10 s Rigidity, Gait ~60–90 s
Closed-Loop	Multiple channels of input biomarkers for continuous analysis	Continuous prediction of brain intent for action	20–50 ms updating

*The use of biomarkers can be described in various approaches, including continuous (i.e., no variation in stimulation except with occasional clinical programming changes over time), and intermittent (i.e., the device is scheduled to have preset amplitude turned on and off at specified intervals). Responsive and adaptive show progressively more flexibility in when to perform stimulation (i.e., triggered by an event or threshold) and adaptive has inherently further flexibility in prolonged stimulation and levels of stimulation when on. Closed loop can apply to any scheme where a feedback signal is used to alter stimulation, but commonly is used in a brain-machine context, in which brain intent (i.e., for an action) is analyzed from multiple channels, then predictions for the next epoch are calculated, with visual or sensory feedback to correct. The chart gives the type of feedback which can be used, the nature of the feedback and time constants to be considered in delivering the feedback*.

## Neuropace and DBS devices

Rather than constant, continuous stimulation (i.e., Figure 1A) DBS for epilepsy implements an intermittent, scheduled stimulation at a preset level, potentially being more effective than constant stimulation (Fisher and Velasco, 2014) (Figure 1B, Table 1). Additionally, phase-dependent stimulation may exert a critical dampening if the response can be sufficiently rapid (Cagnan et al., 2017). In contrast, the Neuropace IPG device implements triggered or responsive stimulation to treat specific, epileptic events detected by a threshold crossing on the input channels (Figure 1C), and this may be less intrusive than constant stimulation if the events or symptoms are uncommon (Morrell and RNS System in Epilepsy Study Group, 2011). Once a pre-determined stimulation level and threshold are set the device both records ongoing brain activity and then triggers the preset stimulation burst based upon threshold criteria, with a lockout preventing overstimulation. Newer Medtronic devices (i.e., Medtronic PC+S and RC+S) also have the capability for lock-out, phase-in timing and adjustable, contingent response to the input signal (Rouse et al., 2011; Afshar et al., 2012; Stypulkowski et al., 2013). The Medtronic RC+S is the more advanced version of the PC+S with additional sensing and stimulation channels (16 vs. 8), rechargeable, improved real-time data access, and ability to connect to 4 4-channel electrodes, available in 2018 for research testing.

## Responsive control

Some disease processes, such as epilepsy, may have long asymptomatic periods between events when stimulation is not required. Rather than subjecting the patient to the potentially deleterious effects of constant stimulation and to avoid chronic circuitry changes that may lead to loss of efficacy, scheduled intermittent stimulation (such as anterior thalamic DBS where stimulation is applied for 20–30 s with an off-time of ~5 min) reduces the amount of unnecessary stimulation by intermittently modulating the network to a less excitable state. However, the parameters of stimulation when “on” are fixed and stimulation occurs in response to detection of a potentially ictal or hyperexcitable state (Fisher and Velasco, 2014). In contrast, the Neuropace system, a responsive system, constantly monitors a set of brain electrodes for activity and then, when intermittently triggered by a critical condition, stimulates the brain to prevent seizure propagation (Morrell and RNS System in Epilepsy Study Group, 2011). Similarly, there are plans to develop a responsive or triggered DBS epilepsy system based on cortical events (Stypulkowski et al., 2014), similar to a prototype Tourette's control system with thalamic stimulation based on occurrence of cortical events (Almeida et al., 2015). In continuous, scheduled, and responsive paradigms, manual parameter adjustments performed by a clinician can be a time intensive process as the stimulus is slowly altered and the outcome is assessed at intervals.

The responsive stimulation in Neuropace implements recording electrodes to “sense” an ictal event via complex internal algorithms in its controller, and turns stimulation on and off only as needed to interrupt a developing seizure (Durand, 2009; Morrell and RNS System in Epilepsy Study Group, 2011). In these systems, upper and lower thresholds trigger the binary on and off states of the system (analogous to the thermostat controls for furnace systems), delivering stimulation when triggered at a predetermined level and with a constant width, based on prior events. This control system would appear to be best suited for diseases with intermittent and unpredictable manifestations, such as epilepsy or Tourette's Syndrome (Morrell, 2006; Okun et al., 2013). The inputs to these control systems may be external or internal, ranging from surface electromyogram (EMG) to scalp electroencephalogram (EEG) to local field potentials (LFPs) (Basu et al., 2013; Priori et al., 2013a). For example, Graupe et al. describe the use tremor-predictive information from surface EMG and accelerometers as the input to an on-off adaptive DBS system in tremor control (Graupe et al., 2010).

## Adaptive and closed-loop control

The integration of more advanced control systems concepts into brain stimulation can be found in the last two types of stimulation–adaptive and closed loop systems. Adaptive stimulation has adjusted stimulation in response to either external signals or an internal or biomarker signal, which has a close relationship to an external symptom. First patented in 1996 (by Michael S John), adaptive stimulation was envisioned for maintenance of “consciousness” in traumatic brain injury patients (US Patent 6066163A, 2/2/1996).

Little et al. investigated short term (externalized) adaptive DBS of the STN for Parkinson's disease based on processing of local LFPs and using an on/off system dependent on beta frequency amplitude, but with dynamic, variable widths of stimulation, hence fitting within the definition of adaptive (Little et al., 2016a,b). Interestingly Little et al. compared continuous, scheduled intermittent (termed “random” in their study), and adaptive systems head-to-head and found statistically significant motor scores, reduction in stimulation time, decreased speech side effects and decreased energy requirements with the adaptive system when triggered by a threshold but not with random stimulation (Little et al., 2013). As noted with later analysis of these and additional data, the adaptive on/off system resulted in much improved efficiency and a modest improvement in efficacy (Tinkhauser et al., 2017). The resultant improvement in clinical motor scores was also corroborated by Rosa et al. in one patient comparing continuous DBS and adaptive DBS, which utilized a continuously varying stimulation amplitude parallel to the beta frequency content (Rosa et al., 2015). Alternatively, Priori et al. implemented recordings of internal LFPs as the input to their adaptive DBS for Parkinson's disease (Priori et al., 2013a).

A scalar adaptive system has also been proposed, using variable amplitude inputs to hone in on a control point, but only tentatively implemented by Rosa et al (Santaniello et al., 2011; Carron et al., 2013; Rehan and Hong, 2013; Rosa et al., 2015). In this concept, a scaled output is provided based on the difference between a setpoint and current state, such that a larger difference from a desired value creates a larger change in stimulation (Figure 1D). A classic control system principle outside of medicine, this slightly more sophisticated variation minimizes the amount of output variable oscillation and time to reach a desired set point, as well as large or complete on/off changes which may be disturbing to the patient.

In contrast to intermittent or adaptive stimulation, closed-loop stimulation depends upon a constant, rapidly updated feedback parameter, such as visual feedback in brain computer (or brain machine) interfaces (BCI/BMI) using constant brain sensing (i.e., as in a motor task) and direct, nearly continuous contingent output for motor control (Lebedev and Nicolelis, 2006; Leuthardt et al., 2006; Patil and Turner, 2008). The update time for BMI systems is typically on the order of ~20 Hz (i.e., ~50 ms for updating the next prediction) (Hanson et al., 2012). Additionally, whereas adaptive stimulation using a DBS device may have only a single setpoint, closed-loop stimulation may enable a wide range of trajectories with continuously adjustable setpoints in a highly dynamic sense, approaching a target and performing a task (Khobragade et al., 2015).

Although the initial work focused on motor disorders, more recently Widge and Sahay also discussed the concept of closed-loop applications in psychiatric disease (Widge and Sahay, 2016), with attention to BCIs as a better, dynamic, real-time source of input for psychiatric disease states. While largely theoretical, early work in this area suggests that closed-loop feedback can remap and alter neural network firing patterns as the BCI training proceeds, allowing the device to depict intention, which may be critical for treatment of a fluctuating disease like psychiatric illness (Widge and Sahay, 2016).

The Medtronic PC+S/RC+S was developed as an open platform for chronic, adaptive brain stimulation, and has been used in a wide range of pre-clinical and clinical applications (Stanslaski et al., 2012; Carlson et al., 2013; Stypulkowski et al., 2013). These include, for example, developing a brain-computer interface in a locked-in patient with amyotrophic lateral sclerosis (Vansteensel et al., 2016), tremor control linked to a wearable device (Herron et al., 2017), brain stimulation for epilepsy (Stypulkowski et al., 2014), and recording biomarkers while stimulating in Parkinson's disease (Quinn et al., 2015; Neumann et al., 2016; Blumenfeld et al., 2017). Further generations of this PC+S device, particularly the new RC+S IPG in development, also offer the advantage of providing full clinical stimulation when not in research mode, which is desirable for intermittent clinical research in human subjects.

## Characteristics of an ideal biomarker

Although closed-loop brain stimulation has tremendous potential, it requires an effective biomarker to serve as the feedback parameter. A biomarker should directly correlate with the clinical symptoms, such that a system could effectively use the biomarker to control a device in lieu of the specific symptoms (i.e., beta band oscillations linked to bradykinesia in Parkinson's disease) (Kuhn et al., 2006, 2009). Thus, changes in the biomarker (i.e., beta oscillations) should clearly and accurately predict alterations in the symptoms associated with the disease. Likewise, as the brain stimulation affects the neural circuits, the biomarker should directly reflect these changes and constantly and dynamically track disease state. Biomarker signals, including beta frequency oscillations in Parkinson's disease as well as further signals under consideration, are also subject to sampling issues (i.e., which site provides optimal signals if several are available), noise from surrounding brain signals obscuring the signal, and in particular signal stability over time (Steiner et al., 2017). For example, cortical pathological signals may overlap with cortical movement signals in some contexts, requiring some form of identification of signals before being used for control. Likewise, highly variable signals may be noted in the same region from different patients, leading to a need for fine tuning and adjustment for individual patients (Kent et al., 2015).

## Biomarkers by disease process or pathology

**Figure 2:**
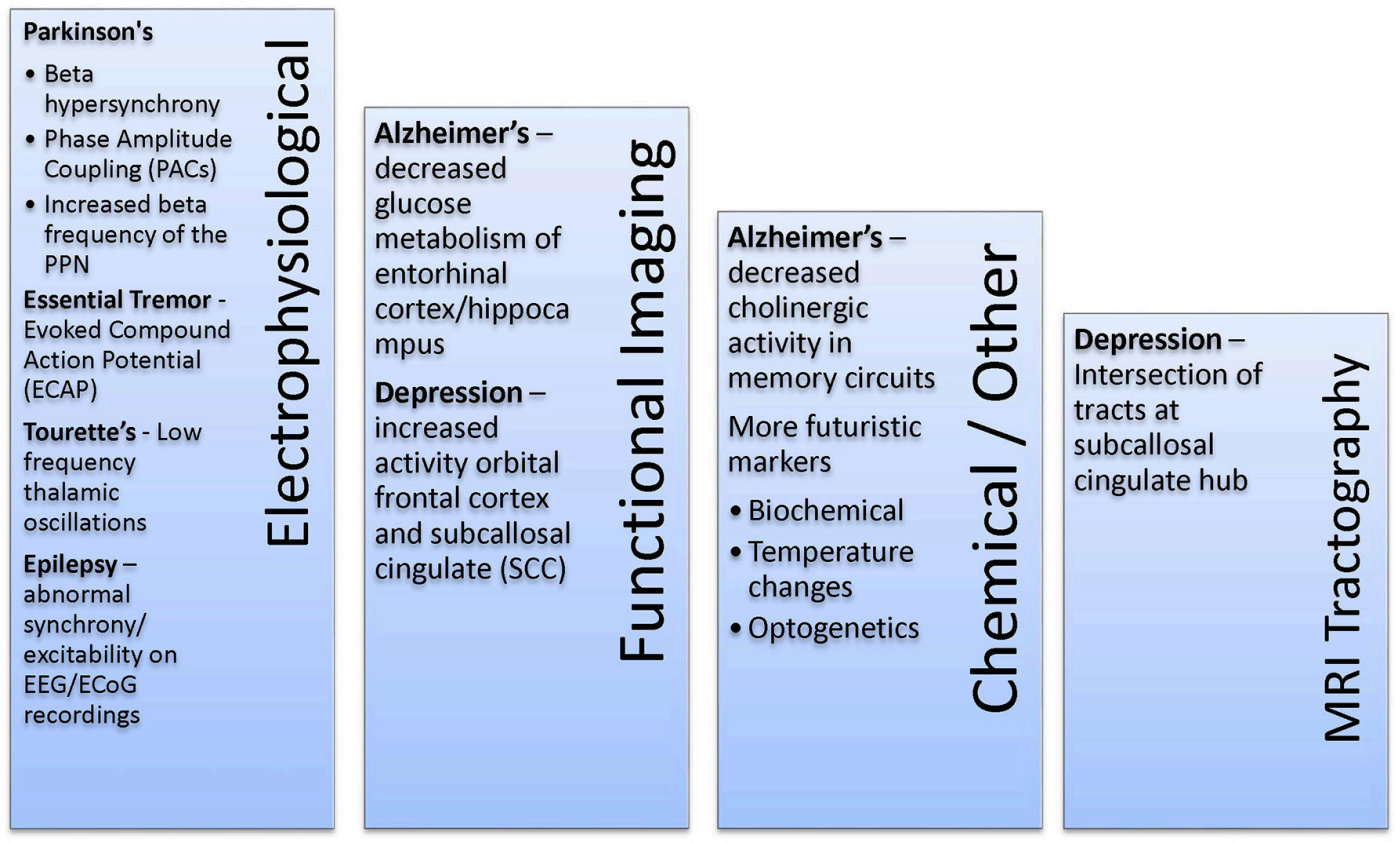
Potential Biomarker by Type. Biomarkers divided into electrophysiological, imaging, and other categories.

We review how biomarkers are implemented by disease category, beginning with epilepsy, in which the Neuropace device is now approved and starting to be more widely used. The next category includes Parkinson's disease and Tourette's syndrome, in which ongoing experiments in humans are starting to demonstrate the potential of biomarkers. Lastly, we discuss conditions in which biomarkers under development may help improve possible brain stimulation approaches, but which have not yet shown clinical utility, including continuous stimulation. These latter conditions include depression and other psychiatric diseases, Alzheimer's disease, and dystonia, for which possible biomarkers have been defined but have not yet been implemented in clinical trials. Our discussion by disease category is tabulated in Table 2. Alternatively, biomarkers and disease states are categorized in Figure 2 by biomarker type.

**Table 2:** Possible targets, affected circuits, and potential surrogates.

**Disease**	**DBS targets**	**Circuit**	**Postulated surrogate**
Parkinson's (PD)	STN, Globus pallidus interna	Motor (niagro- striatia-pallido- cortical circuits)	Beta hyper synchrony, Phase Amplitude Coupling (PACs)
(PD- freezing of gait)	Pedunculopontine nucleus(PPN)	White matter tracts between PPN and motor circuits	Increased beta frequency or cholinergic neuron action potentials
Essential tremor	Vim nucleus (of thalamus)	Motor	Evoked compound action potential (ECAP)
Alzheimer's disease	Fornix, entorhinal cortex, hippocampus, cingulate, precuneous, frontal cortex	Cognitive and Memory circuits	Volumetric analysis and glucose metabolism changes on PET/SPECT, particularly entorhinal cortex and hippocampus; cholinergic degeneration
Tourette's	Centromedian nucleus of thalamus and GPi	Motor/limbic	Low frequency thalamic oscillations resulting in lack of thalamocortical inhibition
Depression	Subcallosal Cingulate (SCC) and Area 25 (medial forbrain bundle), nucleus accumbens, habenula	Limbic	Tractography intersection hub of three fiber bundles near SCC; increased activity in orbital frontal cortex/sec
Epilepsy	Anterior thalamic nucleus, CM thalamus, localized seizure focus	Various	Abnormal synchrony and excitability noted on EEG, ECoG and depth electrodes

*These sites are show compiled for the various disease processes as outlined by this review. CM, centromedian nucleus (of the thalamus); PET, positron emission tomography; SPECT, single photon emission computed tomography; STN, substantia nigra; EEG, electroencephalogram; ECoG, electrocorticography*.

## Epilepsy

Neurostimulation for medication resistant epilepsy can take the form of peripheral nerve stimulation like vagal nerve stimulation (VNS), customized electrodes using the responsive Neuropace system, and anterior thalamic DBS (Fisher and Velasco, 2014). VNS and responsive Neuropace devices are approved in the USA and thalamic DBS is approved in a number of other countries, although not the US at the time of writing (Fisher and Velasco, 2014). Neuropace stimulation in response to activity detected with a strip or depth electrode adjacent to a previously identified epileptogenic zone demonstrated an improvement in relative seizure frequency of about 21% (specifically, 38% reduction in the treatment group vs. 17% reduction in the sham group) (Morrell and RNS System in Epilepsy Study Group, 2011). Of course, the epileptogenic zone and abnormal brain networks involved with seizure origination and spread must be identified to inform electrode placement, and the fact that an event or seizure must occur for stimulation to begin are limiting factors for responsive stimulation in epilepsy. However, Halpern et al. noted a progression of EEG cortical activity up to 7 h prior to seizure onset, and these pre-ictal changes could represent a future feedback signal for responsive stimulation initiation (Halpern et al., 2008). There are many limitations to implementation of the Neuropace device, including localization as to where to specifically place both sensing and stimulation electrodes, how to detect pre-ictal events sufficiently far enough in advance of a seizure so that stimulation may be subconscious and undetected by the patient, and what stimulation paradigms may actually prevent network oscillations from building up to an ictal event.

Anterior thalamic DBS may modify overall frontal lobe networks to reduce seizure susceptibility, through scheduled intermittent stimulation (Salanova et al., 2015). The pulse repetition frequency of DBS is very important as incorrect frequency leading to EEG synchrony (rather than suppression) can lead to increased seizures rather than seizure control (Durand, 2009). The anterior nucleus of the thalamus (ANT) has shown long term significant seizure frequency reduction in the SANTE trial using intermittent scheduled stimulation (Salanova et al., 2015). DBS placement in the ANT is often guided by microelectrode recording and characteristic stimulation effects on scalp EEG in the frontal lobes (Halpern et al., 2008), which could be used as an intraoperative marker for effective location of the DBS electrodes.

These clinical studies build upon numerous pre-clinical research studies focusing on the effects of stimulation inhibiting seizures, particularly since brain stimulation can also commonly induce seizure activity (Durand, 2009). For example, a landmark pre-clinical study demonstrated the ability to control epileptic events from skull (extracranial) stimulation, using implanted brain electrodes as the closed loop feedback circuit for measuring ongoing cerebral hyperactivity (Berenyi et al., 2012). The advantages of the extracranial approach for seizure control is that excitability in a wide swath of brain can potentially be controlled, whereas the focal stimulation afforded by the Neuropace device can in some instances be insufficient for seizure control.

## Parkinson's disease

Parkinson's disease (PD) is a chronic, progressive movement disorder that affects ~0.9 million people in the United States (Ascherio and Schwarzschild, 2016; Lee and Gilbert, 2016). DBS for PD is commonly applied to the STN and, equivalently, to globus pallidus internus (GPi) (Benabid et al., 2009; Williams et al., 2014; Delong and Wichmann, 2015), though the three year follow-up of the NSTAPS study has recently confirmed improved efficacy for STN (Odekerken et al., 2016). Although ~75% of patients get symptom relief with DBS (Odekerken et al., 2016), there remains considerable patient to patient variation in outcomes in spite of current electrode localization and placement strategies, possibly since the pathways and pathophysiology of the disease itself are not fully understood (Whitmer et al., 2012). In some cases, insufficient treatment with a single DBS electrode may be addressed with an additional electrode. For example, instability and freezing of gait (FOG) remain significantly debilitating and causes of fatal falls, hip fractures, and pneumonia in Parkinson's patients (Aminoff et al., 2011). STN DBS leads to improvement of these axial gait symptoms but not in all patients (Chenji et al., 2017; Schlenstedt et al., 2017). Further, one study implemented the measurement of PPN evoked potentials to help guide GPi DBS stimulation in a closed loop configuration, altering the GPi input based on the PPN output (Morita et al., 2014); however, conclusions are not yet available as this is a study in progress. Another consideration for dual electrode recordings is the use of STN+GPi, which has primarily been suggested for improving persistent dyskinesias after STN DBS (Sriram et al., 2014; Cook et al., 2015; Matias et al., 2016). Dual electrodes may also facilitate stimulation on one electrode and recording on another with reduced intrinsic stimulation artifact on the sensing channels.

### Beta band oscillations

Synchronization of cortex, basal ganglia, and thalamus results in spontaneous oscillatory activity in PD in the beta band, at around 13–30 Hz, spreading throughout the cortico-basal network (Wingeier et al., 2006; Whitmer et al., 2012), and this activity is thought to be a marker of PD state in animals and humans (Kuhn et al., 2006, 2009). Hypersynchrony declines after therapeutic doses of dopaminergic medication and likely DBS at an adequate dose (Weinberger et al., 2006; Little and Brown, 2012). Such synchrony may represent a biomarker that can be quantified to guide lead placement and to determine optimal DBS parameters (Whitmer et al., 2012; Lozano and Lipsman, 2013). These beta band responses can be recorded both from motor cortex [i.e., electrocorticography (ECoG)] (De Hemptinne et al., 2015) as well as directly from STN DBS contacts (Gmel et al., 2015; Brocker et al., 2017).

The hyperdirect pathway carries excitatory input from the motor cortex to the STN (Delong and Wichmann, 2015). Recently Whitmer et al. recorded subdural ECoG and STN LFPs (in a clinical study during DBS placement in 13 humans) from both within and adjacent (dorsal) to the STN to evaluate attenuation of hyper-synchrony following STN activation (Whitmer et al., 2012). Beta band attenuation varied positively with DBS amplitude at clinical treatment levels, suggesting that beta band measurements may provide a biomarker of PD treatment effect for bradykinesia (but not necessarily tremor). However, there was enhanced attenuation of hyper-synchrony by DBS within the central STN as opposed to leads placed dorsal to the STN (Marsden et al., 2001; Mackinnon et al., 2005). This highlights the possibility that different symptoms within the same disease process may involve different nodes within the neural network, or possibly variable neural networks. Further, the ECoG recordings confirmed that the beta synchronization was also present in the premotor and motor regions, a possible indication that the STN is a node between linked regions in the cortical basal ganglia circuit. However, a direct correlation with PD symptom improvement remains a critical requirement (Whitmer et al., 2012).

### Entropy

In addition to reduction in synchronization, effective DBS leads to regularization in neuronal firing patterns (Hashimoto et al., 2003; Bar-Gad et al., 2004). For example, the highly oscillatory, pathological firing patterns present in PD may be replaced by DBS-evoked action potentials with regular patterns that can act as an innocuous signal (Dorval et al., 2010). This regularity can be quantified via the firing pattern entropy, which is calculated from the inter-spike intervals of single unit recordings. In the rodent GP and substantia nigra pars reticulata (SNr), entropy increases with parkinsonism and decreases back to near healthy levels with effective DBS (Dorval et al., 2008, 2015; Dorval and Grill, 2014; Anderson et al., 2015).

### Directed information

Along with firing pattern entropy, directed information transfer between neurons also correlates with DBS efficacy (Dorval and Grill, 2014; Anderson et al., 2015; Dorval et al., 2015). Directed information measures the degree of influence that a recorded single neuron has on another neuron (Anderson et al., 2015). In rodents and non-human primates, directed information increases with Parkinsonism between the SNr and ventral anterior thalamus and between the GP internus and GP externus, respectively. In both cases, directed information is then reduced with effective DBS (Anderson et al., 2015; Dorval et al., 2015). Although an increase in directed information with Parkinsonism might be mistaken as beneficial, it most likely indicates the pathological hyper-synchrony observed in the basal ganglia, with each neuron passing on redundant information. A reduction in directed information with DBS could thus indicate a break in this hyper-synchronous state and a return to independence between information channels. Although based on firing of single neurons, directed information may or may not be a feasible biomarker in humans, unless the unit firing can be translated into either a spontaneous or evoked potential measurable with more stable electrodes.

### Phase amplitude coupling

Using ECoG recorded over the motor cortex, De Hemptinne et al. observed that DBS reduces exaggerated primary motor (M1) PAC between beta oscillations and higher frequency superimposed oscillations characteristic of PD (De Hemptinne et al., 2013, 2015). Normal cortical function also involves the presence of PAC, possibly coordinating timing of neuronal activity between and within cortical areas as necessary for task performance (Canolty et al., 2006; Canolty and Knight, 2010; Yanagisawa et al., 2012). The increased PAC in PD possibly reflects neurons constrained into an inflexible pattern by PD pathophysiology, leading to rigidity and bradykinesia (Moran et al., 2008; De Hemptinne et al., 2013). DBS normalizes elevated PAC, both at rest and during a motor task (De Hemptinne et al., 2015), and PAC may be a sensitive biomarker to measure both the Parkinsonian state as well as the effectiveness of therapy for these symptoms (De Hemptinne et al., 2015). Unfortunately, the presence of PD tremor alters this measure and voluntary motion also can change PAC. However, the advantages of minimal stimulation artifact in the ECoG recording electrode could potentially be applied to other neurological or psychiatric disease states (De Hemptinne et al., 2015). Overall, whether PAC can be effectively translated into a useful and consistent biomarker, separable from normal motor function, has yet to be demonstrated.

### Evoked potentials and oscillations

Evoked responses from stimulating and recording via the same DBS lead in patients during DBS surgery (using different contacts) can provide neural circuit-specific insight into functional activation of the basal ganglia through STN–GP synaptic interactions, particularly through a comparison of responses to stimulation at effective (i.e., ~130 Hz) and ineffective (~10–40 Hz) stimulation frequencies (Grill et al., 2015). Further, gamma band oscillations (i.e., 60–90 Hz) may indicate hyperkinetic PD symptoms, such as dyskinesias, as a biomarker separate from beta band oscillations (Swann et al., 2016). Any of these electrophysiological markers could theoretically be inputs to closed-loop systems or markers of disease-treatment response in a slowly progressive disease, such as PD.

### Summary of PD

Parkinson's disease (PD) is a progressive disease with varying presentations, including motor and non-motor symptoms. Conventional DBS targets (i.e., STN, GPi) focus on symptomatic relief of motor symptoms, although STN DBS does show also consistent improvement of midline and non-motor symptoms, particularly FOG. There is also the possibility of multifocal targeting with two or more DBS electrodes within known nodes (i.e., STN+GPi, for example), but this configuration will have the added complication of how to adjust cooperatively parameters from the additional recording/stimulation contacts. It also further highlights the need for brain modeling to understand circuitry and biomarkers to assist in automatic adjustment (Butson et al., 2011; Butson and Mcintyre, 2015). The potential for both a motor target and a cognitive target to treat a single disease process could also be possible with multiple electrodes (Lozano and Lipsman, 2013). The recent progress with even short-term adaptive systems using an on/off controller shows that beta band oscillations may be a suitable biomarker, particularly for particularly rigidity and bradykinesia (Little et al., 2016a; Tinkhauser et al., 2017). There are many avenues to test these possible biomarkers in initial clinical trials, including intraoperative testing, using percutaneous wires after surgery for several days, or using one of the implanted sensing/recording devices in small clinical cohorts.

## Tourette syndrome

Tourette syndrome (TS) is an idiopathic neuropsychiatric disorder defined by motor and phonic tics and often associated with psychiatric disorders, such as attention deficit hyperactivity disorder (ADHD) and obsessive-compulsive disorder (OCD) (Leckman, 2002; Maling et al., 2012; Almeida et al., 2015). For the small number of patients whose symptoms do not resolve prior to adulthood, the tics can become treatment refractory and debilitating (Jankovic and Kurlan, 2011; Maling et al., 2012; Almeida et al., 2015). DBS for TS first involved bilateral lead placement in the centromedian nucleus of the thalamus (Vandewalle et al., 1999). Currently, it is theorized that dopaminergic neurons contribute to a dysfunctional circuit that leads to decreased cortical inhibition (Gilbert et al., 2004; Almeida et al., 2015) and excessive inhibition of basal ganglia output to the thalamus (Mink, 2001). Current DBS targets include the centromedian-parafascicular complex of the thalamus with the nearby anterior portion of the ventralis oralis nucleus. As well, the efficacy of intermittent / scheduled as compared to continuous stimulation is currently being evaluated (Okun et al., 2013).

Recordings from the thalamic nuclei of anesthetized TS patient undergoing DBS revealed low frequency firing in bursts (Priori et al., 2013b). Awake, these frequencies seem to match with the clinical phenotype and seem preferential for tics as opposed to the OCD manifestation of TS. Maling et al. studied 5 patients implanted with the Neuropace device using cortical ECoG strips, CT-MRI fusion and intraoperative microelectrode recordings to delineate their anatomic CM target (Maling et al., 2012). Over time, the best responders to adaptive stimulation of the thalamus following bursting in the ECoG demonstrated increased gamma activity and return to higher frequencies with the best symptom relief correlating with synchronization within a single oscillatory frequency. Furthermore, GPi low frequency oscillations preceded EMG recordings of the tic by 50–2,000 ms, a potential foreshadowing of tic activity based on the suspected anatomical circuit (Priori et al., 2013b). For patients with OCD comorbid to their TS, the anterior limb of the internal capsule and nucleus accumbens is another anatomical target. LFP recordings demonstrate high beta frequency oscillations, another possible physiological marker of OCD, and a speculative precursor to low frequency thalamic oscillations (Priori et al., 2013b). These changes in oscillatory activity could also provide an adaptive trigger for preventing motor tics through demand DBS stimulation.

## Essential tremor

Stimulation of the ventral intermediate nucleus of the thalamus [Vim] is useful for control of essential tremor, which affects ~7 million people in the United States (Deuschl et al., 2011). Spontaneous or evoked responses may be directly recorded from the DBS lead (Afshar et al., 2012). Kent et al. investigated the evoked compound action potential (ECAP) intraoperatively within Vim as a potential surrogate marker in thalamic DBS (Kent et al., 2015), with the ECAP arising from synchronously-activated neural elements (likely axons) near the lead (Kent and Grill, 2012). While recording from two non-stimulating contacts of the lead, stimulation parameters and tremor measured via accelerometer were correlated with neural activation assessed from the ECAP (Kent et al., 2015). High frequency 130 Hz DBS reduced essential tremor, while 10 Hz DBS worsened tremor. During high frequency DBS, a monotonic relationship between voltage and tremor was noted up to an optimal voltage; however, ECAP signal amplitudes varied as much as an order of magnitude between subjects. The study also suggested that cerebellar afferents were the primary determinants of the ECAP response (Kent et al., 2015). Lastly, it was noted that as more glial scar or electrode conditioning with stimulation occurred around the leads with continued chronic DBS (measured during IPG replacements), the stimulation artifact in the ECAP recording increased, potentially hampering long-term applications (Henderson et al., 2002; Kent et al., 2015). A consistent evoked response with consistency across patients has proven elusive, however.

Another concept to improve the signal to noise ratio during Vim DBS would be to record the evoked field potential within postsynaptic areas anterior to Vim, where the cerebellothalamic fibers have their synapses (in VOP particularly) (Gallay et al., 2008). This would involve recording from a more anterior DBS electrode, which has typically been placed for improved tremor control in difficult cases (Foote and Okun, 2005). The advantages of a second electrode include the possibility that all contacts on the primary electrode could be used for stimulation (as opposed to sharing stimulation and recording contacts) and that there would be improved isolation from the stimulation artifact, similar to recording from the isolated ECoG motor cortex strip electrode (Kent and Grill, 2012; Almeida et al., 2015).

External signals from wearable devices, such as accelerometers, may also provide direct and dynamic feedback on tremor intensity, and can be potentially included in closed-loop control (Herron et al., 2017). For example, Cagnan et al. (2017) developed a prototype system for phase-specific stimulation, linking the measured phase of tremor to the DBS IPG for intermittent stimulation. This worked well for tremor control in some patients and could potentially improve the efficiency of stimulation with reduced IPG output. Further, Slavin et al. developed a tremor prediction and control algorithm for on/off adaptive control of tremor based on muscle contraction and accelerometry (Shukla et al., 2012; Basu et al., 2013). Potentially, the internal accelerometer with the Medtronic PC+S device could also function to detect tremor within the upper extremities, though differentiating one arm from the other may be difficult (Afshar et al., 2012; Stanslaski et al., 2012).

## Depression

Deep brain stimulation (DBS) for treatment resistant depression (TRD) has developed over the past 10 years (Crowell et al., 2015) with targeting of multiple sites, including subcallosal cingulate (SCC) white matter or Brodmann area 25 gray matter, ventral capsule/ventral striatum, nucleus accumbens, inferior thalamic peduncles, medial forebrain bundle, and the lateral habenula (Mayberg et al., 2005; Schlaepfer et al., 2013; Riva-Posse et al., 2014; Crowell et al., 2015). While small trials demonstrated efficacy even at long-term time points (Holtzheimer et al., 2012), larger industry sponsored trials, such as BROADEN (St. Jude) did not (Dougherty et al., 2015). Invasive electrophysiological recordings from reward and mood circuitry are far less common than those from movement centers (Lozano and Lipsman, 2013). However, a recent neurophysiological study combined SCC DBS in depression with (non-invasive) EEG pre- and post-operatively and observed that coherence of pre-operative frontal lobe theta EEG signal across multiple recording sites predicted a better response to DBS treatment (Broadway et al., 2012).

The SCC is the most studied site, initially chosen following imaging data showing changes in the SCC white matter in response to standard antidepressant treatment (Mayberg et al., 2000; Seminowicz et al., 2004; Mayberg, 2009). Lead placement in DBS studies of SCC was initially anatomically guided to white matter (Mayberg et al., 2000; Holtzheimer et al., 2012). However, therapeutic responses to DBS differed among those studied, and anatomical location did not predict those who would and would not respond (Hamani et al., 2009). Further PET studies, fiber tractography, DBS activation volumes and probabilistic tractography subsequently supported a theory that SCC is part of a larger circuit of multiple afferents to the frontal lobes as well as fibers of passage in this area (Hartmann et al., 2015), which may prove equally important as SCC in depression modulation (Butson et al., 2007; Hamani et al., 2009; Lujan et al., 2013). Thus, anatomical localization alone appears unable to predict which specific white matter tract is being stimulated and which frontal lobe target receives the stimulation. To overcome this limitation, Riva-Posse et al. observed that clinical response to SCC DBS was linked to proximity of the intersection of three fiber bundles: (1) bilateral forceps minor of the anterior corpus callosum, (2) bilateral cingulum bundles connecting ipsilateral SCC to rostral, dorsal anterior, and mid-cingulate cortices, and (3) medial branch of the uncinate fasciculus connecting the subcallosal cingulate and medial frontal cortex rostrally and subcallosal cingulate to the nucleus accumbens and anterior thalamus (Riva-Posse et al., 2014). Prospective trials of this intersection as well as directional steering and specific evaluation of evoked responses to DBS in the various frontal lobe areas (i.e., with ECoG) to evaluate each of the components of the fiber bundles would be helpful in understanding if indeed tractography provides a better target for depression modulation (Martens et al., 2011; Riva-Posse et al., 2014).

Intraoperative testing is challenging in DBS for TRD as patient response is highly variable and personal. Autonomic effects (tachycardia and increases in skin conductance) appear to correlate reproducibly in the OR with appropriate lead placement via tractography as described above (Riva-Posse et al., 2014) and may provide reliable biomarkers of post-operative response and efficacy (Crowell et al., 2015). As another perioperative marker of clinical efficacy, EEG frontal theta changes noted 1 month after DBS appear to predict 6 month response to DBS (Broadway et al., 2012). Further physiological biomarkers reflect subcortical limbic changes in depression (Neumann et al., 2014). Each of these quantifiable changes could be assessed as potential biomarkers with respect to depression and validated as potentially relevant biomarkers.

## Dystonia

Primary dystonia is an uncommon movement disorder (~16–17/100,000 population) characterized by abnormal, repetitive muscle contractions and postures (Steeves et al., 2012; Williams et al., 2017). DBS to improve motor features of focal and diffuse dystonia is FDA approved in the USA, though only as a humanitarian device approval due to the low numbers and decreased efficacy, which is equivalent to an orphan drug approval. Of the dystonias, focal dystonia (i.e., cervical dystonia or torticollis and writer's cramp are examples) is the most common, followed by genetic (i.e., DYT1) generalized dystonias (Williams et al., 2017). Unlike Parkinson's disease or tremor, the time course to onset with changes in stimulation in dystonia can take beyond seconds or minutes, usually taking weeks to months to reach full effect. The onset effect of brain stimulation in dystonia is difficult to predict, suggesting possible plasticity or synaptic changes associated with stimulation, leading to slow improvement in symptoms. However, Barow detected suppression of low frequency pallidal activity during treatment of phasic, dystonic movements, and this may improve our understanding of this slow time to onset as a possible biomarker (Barow et al., 2014). The slow dynamic effect also creates difficulties with developing an adaptive or closed-loop system due to unpredictable timing and might suggest only very slow (weeks to months) adjustments (Wang et al., 2016). However, there are several studies analyzing the differences in subcortical recorded potentials between Parkinson's disease and dystonia, both as initial evidence to discover how the circuit changes underlying these diseases differ and to develop biomarkers of dystonia (Silberstein et al., 2003; Neumann et al., 2015; Wang et al., 2016). For example, Wang et al. found no difference in biomarkers between the two diseases (Wang et al., 2016) whereas Geng et al. suggested more specificity (Geng et al., 2017), indicating that biomarkers may in some instances be useful disease indicators.

## Alzheimer's disease

As a predicted future health burden without good current treatment, Alzheimer's Disease (AD) may theoretically respond to brain stimulation (Hardenacke et al., 2013b; Hescham et al., 2013; Sharma et al., 2015). Novel techniques, such as DBS of the fornix were developed as an offshoot of hypothalamic stimulation when it was noted that fornix stimulation resulted in improved recall (Laxton et al., 2010). It should be noted that possible loss of functional integrity of the fornix in AD may limit the effectiveness of fornix stimulation (Mielke et al., 2012). However, it is difficult to test the efficacy of DBS applied to fornix stimulation intraoperatively so biomarkers may be very helpful to confirm appropriate electrode location as well as to set DBS parameters.

Although fornix stimulation in clinical trials has shown limited clinical efficacy (Ponce et al., 2015; Lozano et al., 2016) the downstream effects of stimulation of the post-commissural fornix (below the anterior commissure) on the mammillary bodies remain unknown. The post-commissural fornix antidromically stimulates the subiculum, since this is the main hippocampal output, resulting in a large hippocampal evoked potentials (Stypulkowski et al., 2014; Sweet et al., 2014). This evoked potential (as recorded from the hippocampus) could provide an excellent biomarker both to adjust fornix stimulation amplitude (currently empirically determined) and to estimate if there is indeed plasticity resulting following a period of fornix stimulation, possibly leading to an on-demand fornix stimulation system to maintain hippocampal evoked responses within a set, optimal range. Although the current clinical application of fornix stimulation does not include the medial septum (just in front of the anterior commissure), additional stimulation of the medial septum could facilitate memory and hippocampal vascular changes through cholinergic enhancement, also resulting in an evoked response within the hippocampus proper (Colgin et al., 2003; Gu and Yakel, 2011). Further, DBS of the nucleus basalis of Meynert, the primary cholinergic region for the cortex, may diffusely enhance cholinergic function and also improve AD, without direct fornix stimulation (Hardenacke et al., 2013a; Sharma et al., 2015). A cholinergic sensory or biomarker within the brain may be required to measure effectively such cholinergic enhancement and to create a dose-response curve. Thus, there are a number of clinical avenues which may enhanced the potential treatment possibilities of brain stimulation for AD.

## Conclusions

Identifying and verifying the usefulness of biomarkers for accurate electrode placement and creating more effective adaptive brain stimulation systems will require considerable additional development, and is clearly subject to significant research and initial clinical trials. Methods for optimal electrode localization and verification of both symptom suppression and physiological efficacy are clearly needed, likely through modulation of appropriate neural networks. Placing stimulating electrodes into defined anatomical targets in the operating room without symptom suppression or intraoperative clinical testing will require better imaging with emphasis on pre-operative tractography and functional MRI as well as use of biomarkers during surgery, as they are developed and validated. Understanding pathophysiology and structural connectivity in individual disease processes will elucidate key pathways and, subsequently, the hubs of electrical activity for optimal sites of stimulation. Spontaneous and evoked potentials, phase-amplitude coupling, and other electrophysiological measures can provide intraoperative data beyond imaging to place leads in locations correlated with more reliable outcomes. Possible neurochemical or other markers may also act as biomarkers to join imaging and electrophysiological measures as suitable signals for stimulation optimization. As external, wearable devices become more widespread and available, further data on motion and tremor may also eventually be included in adaptive control on a long-term basis if these data can be communicated effectively to an internal device (Roy et al., 2013; Shull et al., 2014; Lieber et al., 2015). Further development in this area has the potential to alter significantly our understanding of the underlying circuitry and pathophysiology of neurologic disease, as well as optimize treatment approaches.

## Author contributions

KH, IC, WG, and DT wrote this manuscript collectively, with KH contributing the initial draft and subsequent significant editing by all of the authors. All contributed to the ideas contained within the manuscript and all contributed at least one major section. All authors have reviewed and edited this final version.

### Conflict of interest statement

The authors declare that the research was conducted in the absence of any commercial or financial relationships that could be construed as a potential conflict of interest.

## Abbreviations

DBS: deep brain stimulation
VNS: vagal nerve stimulation
TBI: traumatic brain injury
aDBS: adaptive DBS
IPG: internal pulse generator
EEG: electroencephalogram
ECoG: electrocorticography
EMG: electromyogram
PAC: phase amplitude coupling
ECAP: evoked compound action potential
LFP: local field potential
MRI: magnetic resonance imaging
CT: computed tomography
BCI/BMI: brain computer/machine interface
GPi: globus pallidus internus
SNr: substantia nigra pars reticulata
STN: subthalamic nucleus
PPN: pedunculopontine nucleus
Vim: ventral intermediate nucleus of the thalamus
SCC: subcallosal cingulate
ANT: anterior nucleus of the thalamus
PD: Parkinson's disease
AD: Alzheimer's disease
TS: Tourette syndrome
TRD: treatment resistant depression
ADHD: attention deficit hyperactivity disorder
OCD: obsessive-compulsive disorder
FOG: freezing of gait.
